# Dr. Huggan, on Hydrophobia

**Published:** 1807-11

**Authors:** A. Huggan

**Affiliations:** Wallingford


### *To the Editors of the Medical and Physical Journal.*

GENTLEMEN,

YOU did me the favour of inserting in a former part of your Journal, a few loose hints on Hydrophobia, which induces me to trouble you with some further observations on the same subject, if you think them worthy of your notice.

It is quite unnecessary to describe the symptoms of hydrodrophobia, or, as it is more properly called, *rabies contagiosa canina*; as these are in general so well known, and so clearly pointed out by most authors who have written on the disease. I pass on, therefore, slightly to touch upon the nature of rabies (for I shall not stop to discuss it at present), whether that it is *inflammatory*, according to Dr. Rush and other respectable American physicians; and which, in some instances, seems to have been countenanced by dissection; or *spasmodic*, according to Dr. Cullen. It is, I believe, the commonly received opinion amongst the best practitioners in this country, that it is a spasmodic disease, accompanied occasionally by inflammatory symptoms, but which do not necessarily belong to it; and in this light I shall consider it here. But it must be observed, that whatever theory of this disease has at any time obtained, and whatever practice, whether corresponding or not, has been adopted, as is confessed by one of the ablest writers on the subject, "no cure has yet been made in any case of true rabies." Hence it is asserted, with great reason, by the same author, "that those diseases resembling, but improperly called, hydrophobia, and which admitted of a cure, arose not from the saliva of a rabid animal, but from apprehension which sometimes excites mania; or resemble those arising from wounds inflicted on nerves, producing irritation, or various degrees of tetanic affection." (Dr. R. Hamilton's Letter on Canine Rabies, in the last number of the Monthly Magazine). And here I cannot help observing, with your able and industrious correspondent Mr. Ward, that Mr. Hicks's case, in your 97th Number, notwithstanding his very candid and elaborate reply, belongs more properly to this description of disease, than to have been one of true rabies.

Rabies is said to be peculiar to the dog and cat kind *only*, and never appears in the human or any other animal, but by communication from theirs (and from no other) in the usual way; or, in other words, by *inoculation*. It is also supposed to be contagious, as the name imports; and, as has been remarked, all other dogs, from natural instinct, avoid the infected one, when he is, in common language, said to be going mad.

Among the remote causes of rabies, are reckoned cold, hunger, dirty feeding, as carrion and the like; and whatever else tends to weaken, or render their bodies irritable.

But I shall leave this part of the subject, and proceed to the discussion of the means that have been most in use, for the prevention, as well as the cure of rabies; and here

I shall

I shall premise, that as the analogy between this disease and tetanus is so obvious, and now so well established, I shall keep it in view as I go along.

It has long ago been ascertained, that the surest way to prevent rabies, is the excision of the bitten part; and, as if this was not of itself sufficient, keeping the wound open for some time afterwards, by stimulating applications. If, however, the *whole* of the bitten part can be extirpated, without leaving a *single point* behind, which had come in contact with the teeth of the rabid animal, surely the end will be fully answered. Where then is the necessity of increasing and prolonging the patient's sufferings, by keeping him in torture afterwards? But if any thing of this kind must needs be applied by way of further ensuring the patient's safety, I much question if the actual cautery is not the easiest as well as the shortest way of doing it. The practice, at best, is altogether unscientific. To reason from analogy, for it is not a matter of *no importance* to the patient particularly. In making the experiment of trying to prevent small-pox by cutting out the inoculated part, as was done by the late John Hunter, it was never dreamt to be necessary to keep the wound open afterwards, as it succeeded well enough without it; here then the cases are exactly similar. But to return to the subject; when the bitten part cannot be cut out with safety (and when that is the case, it ought not to be attempted, for it cannot possibly do any good, unless the *whole* of it is taken away); we must try to destroy it, by applying *argentum nitratum* to the surface of the wound; or by that or some other means, endeavour to excite such a degree of inflammation in it, as will answer the purpose as effectually. It will be proper, however, in the first place, to lay the bitten part freely open in every direction; so that after washing it well, till the bleeding ceases, and wiping it, we may be able to use the *caustic* with more ease and certainty. Here, however, it may be remarked by the by, that when the wound is very deep, or much lacerated, and cannot be laid open with the freedom that may be necessary, argentum nitratum is a very uncertain application; as some on whom it has been used, under such circumstances, have woefully experienced. What strikes me as more likely to answer the purpose as effectually, is a strong solution of muriated mercury poured into the wound, and which would penetrate into *every part* of it, so as to corrode the whole surface of it, and at the same time excite in it a sufficient degree of inflammation. The patient might, at the same time, be directed to take a drachm drachm of bark three or four times a day. Other articles have also been employed with the same intention, such as cantharides, hydrargyrus, ntr. ruber, strong mercurial ointment, &c.; and a respectable writer advises to fill the wound with gunpowder, and set it on fire.

Another means of preventing, or at least of lessening the danger, is "washing the wound *immediately* and perseveringly with water," as recommended by Dr. Wall * of Oxford, in your 98th Number; and I have no doubt if this could be done the instant the bite is inflicted, but that the poison would be so diluted as to lose its activity entirely, as we know this to happen sometimes to the variolous or the vaccine fluid, when water has been incautiously added, for the purpose of softening it. And I myself have heard persons boast of having escaped venereal infection, by washing with water *immediately* after, when they have known that they deserved it: here the cases are nearly alike. To return, if the water, after thus first washing and cleansing the wound, is poured upon it from a tea-kettle, spouted cup, or the like, till the part is totally benumbed, or in other words, till its *life* or irritability is suspended, which will happen if the process is continued long enough, the end would, in all probability, be answered without farther trouble. However, as the disease to be dreaded is so certainly fatal, few, I believe, would be satisfied with so simple means as this, except with timid people, who will submit to no other. Another way, somewhat similar to this, and of which I have somewhere read, is the *sucking* the wound, according to an ancient practice in armies, when warfare was carried on with poisoned weapons; this, however, like the other, ought only to be preparatory to the employment of means more certainly efficacious.

The only other means of prevention which I have heard of, that is likely to answer the purpose, is a *mercurial* course; and this is supported not only by analogy, in regard to tetanus and syphilis, but has been confirmed by repeated experience; and of which we have another

---

* Whose signature, by mistake, is made to be M. Hall, instead of M. Wall; which lessens the weight of the communication. This Gentleman, who has, for many years, been one of the principal ornaments of the Healing Art in this neighbourhood, is not more admired for his extensive practical knowledge, than for his very liberal and gentlemanlike conduct towards such of his professional brethren, as are occasionally associated with him in practice.

striking striking instance in Number 103 of your Journal, by Dr Harrison. And here let me remark, by the way, if any of the wonder-working nostrums, that have been so often puffed off as never-failing preventatives, have ever had that effect, it must have been owing to some portion of mercury which they have contained, as we know it to be a copious ingredient in some of the most noted of the kind in our own days.

I come now to the CURE, the most discouraging and almost hopeless part of the task; as that, alas! has been attempted in vain by the ablest men in all ages: I shall, therefore, but briefly touch upon what has been, or may be, done towards it, with any thing like a prospect of succeeding.

Rabies, as it appears in man, is, I believe, sometimes preceded by disagreeable or painful sensations about the bitten part, which is said now to begin to inflame, and break out afresh. I mention this for the purpose of asking, if even at this period something *effectual* could not be done, by *powerfully* inflaming, or otherwise irritating the bitten part. But we are seldom afforded an opportunity of even making the trial, as patients, in these cases, do not always have recourse to medical assistance, until the disease has appeared in all its horrors: then the most prompt and decisive measures *only* are likely to avail us any thing. Now, all the circumstances of the disease being considered, nothing appears to me better adapted for answering our purpose in the first instance, than the

COLD AFFUSION, as suggested by Dr. Fothergill (Number 96); and as it is likely to be the readiest means in our power, it ought to be used without a moment's delay, and repeated as often as may be necessary.

SUBMERSION, which is akin to it, notwithstanding the unfavourable issue of Dr. Harrison's case, is not, in my opinion at least, undeserving of further trial. It is to be regretted that the Doctor and his learned friend, had not had an opportunity of examining the body of their patient after death, as the result might, perhaps, have proved more satisfactory than we might be apt to have concluded.

COLD BATHING, which has been found of service in tetanus, especially when it has been used, as directed by Dr. Currie, may probably have the same good effect in this disease. "The administration of it, is sometimes by bathing the person in the sea; or more frequently by *throwing* cold water from a *bason* or *bucket* upon the patient's body, and over the whole of it." (Cullen's First Lines, MCCLXXX).

WARM

Warm Bathing, which is but an ambiguous remedy in tetanus, is not likely to be of more benefit in rabies.

Mercury is the next article in point of importance, and should be used *unsparingly*, both externally and internally, so as to excite a *salivation*, if possible. By this means tetanus has sometimes been cured.

Opium naturally presents itself to the mind here: but if we expect to do any good with it, it must be exhibited in much larger doses than we are accustomed to in common practice; and as its effects do not continue long in the system, we should of course repeat it frequently. At the same time I must acknowledge, that I have had frequent opportunities of seeing opium administered in tetanus, and to a very great extent, in every form in which it can be used; but I cannot add, with the effect that might have been expected.

Musk. In one of the last cases of tetanus, which fell under my own care while in the West-Indies (that of a Negro boy, about 13 years of age), the disease was evidently cut short by musk (conjoined with opium and the volatile alkali), given in a dose of 50 or 60 grains, and repeated every two or three hours.—In rabies it may have a good effect.

Digitalis is suggested as worthy of attention at the same time. I had an opportunity of seeing it administered to an American Negro, in the Naval Hospital at Plymouth, with tetanus, by Mr. Fuge, the senior surgeon (to whom I am indebted for several other useful practical hints) and apparently with benefit.

Tobacco, as an astispasmodic, may be of some service in rabies; and, as it can be administered with equal certainty of producing its effects in form of glyster, as in any other way, it is certainly an argument in its favour, especially if deglutition is either impaired or interrupted; and it has this further recommendation, that it generally proves sudorific at the same time.

Electricity has long appeared to me a very likely means of being of great use in the treatment of this disease; and as it has, of late, been recommended from a quarter that will bring it into further credit, I need not add, that it ought not to be omitted.

Arsenic, which is a very powerful sedative, however its good effects in the cure of agues, chronic rheumatism, &c. are to be explained, is also recommended to be used, by the same respectable authority. I shall only observe further respecting it, that I know from my own *personal*

as as well as other experience, that such a dose of it as will produce an obvious effect on the constitution (and any article that does not, is certainly inadmissible here), is not free from danger.

Blistering has been suggested with some degree of plausibility. It "has been formerly employed in this disease (tetanus); but several practitioners assert, that blisters are constantly hurtful." (Ibid, mcclxxviii).

Oil is recommended to be used both internally and externally; but on what rational grounds, I am at a loss to conjecture.

Bark and Wine, which have been given with advantage in some cases of tetanus, will, as appears to me, be employed with better effect, when the disease is moderated (if happily that can be effected), than during its violence.

Vinegar is said to have been given to the extent of two or three pints a day in rabies, but with what effect I have not learned; nor do I know on what authority.

Bloodletting, which one would hardly think of in treating a case of rabies, is said by Dr. Rush to have been beneficially employed here; and in robust habits, may do no apparent harm; but, if I may be allowed to judge of its probable effect, from what I have seen to follow its use in tetanus, it will be better omitted. I intended here to have noticed the fondness for *evacuations*, that seems to pervade the American practice of medicine; but having already spun this Letter to a greater length than I expected, I shall now conclude with a hope, that the foregoing observations may not be unworthy the perusal of the junior part of your readers. As to those who have had more experience in the treatment of it than I can boast of, as I have yet so much to learn respecting it myself, I shall be gratified by further communications on the subject—

Si quid novisti rectius, candidus imperti.

But after all our speculating (for it can hardly be called experience), upon a cure in this most melancholy of all diseases, I must acknowledge that the prospect is not very flattering.

I am, &c.

A. HUGGAN, M.D.

*Wallingford, October* 10, 1807.

*Addenda*

